# PCR-based landmark unique gene (PLUG) markers effectively assign homoeologous wheat genes to A, B and D genomes

**DOI:** 10.1186/1471-2164-8-135

**Published:** 2007-05-30

**Authors:** Goro Ishikawa, Junichi Yonemaru, Mika Saito, Toshiki Nakamura

**Affiliations:** 1 Tohoku National Agriculture Research Center, Morioka, Iwate, 020-0198, Japan

## Abstract

**Background:**

EST-PCR markers normally represent specific products from target genes, and are therefore effective tools for genetic analysis. However, because wheat is an allohexaploid plant, PCR products derived from homoeologous genes are often simultaneously amplified. Such products may be easier to differentiate if they include intron sequences, which are more polymorphic than exon sequences. However, genomic sequence data for wheat are limited; therefore it is difficult to predict the location of introns. By using the similarities in gene structures between rice and wheat, we developed a system called PLUG (PCR-based Landmark Unique Gene) to design primers so that PCR products include intron sequences. We then investigated whether products amplified using such primers could serve as markers able to distinguish multiple products derived from homoeologous genes.

**Results:**

The PLUG system consists of the following steps: (1) Single-copy rice genes (Landmark Unique Gene loci; LUGs) exhibiting high degrees of homology to wheat UniGene sequences are extracted; (2) Alignment analysis is carried out using the LUGs and wheat UniGene sequences to predict exon-exon junctions, and LUGs which can be used to design wheat primers flanking introns (TaEST-LUGs) are extracted; and (3) Primers are designed in an interactive manner. From a total of 4,312 TaEST-LUGs, 24 loci were randomly selected and used to design primers. With all of these primer sets, we obtained specific, intron-containing products from the target genes. These markers were assigned to chromosomes using wheat nullisomic-tetrasomic lines. By PCR-RFLP analysis using agarose gel electrophoresis, 19 of the 24 markers were located on at least one chromosome.

**Conclusion:**

In the development of wheat EST-PCR markers capable of efficiently sorting products derived from homoeologous genes, it is important to design primers able to amplify products that include intron sequences with insertion/deletion polymorphisms. Using the PLUG system, wheat EST sequences that can be used for marker development are selected based on comparative genomics with rice, and then primer sets flanking intron sequences are prepared in an interactive, semi-automatic manner. Hence, the PLUG system is an effective tool for large-scale marker development.

## Background

Chromosome maps of higher plants were originally constructed by analyzing markers obtained from differences in qualitative traits, such as seed shape or cotyledon color. Although these maps were rather sparse because the number of traits that could serve as markers was limited, they were effective in determining the distance between and order of loci related to these traits. In the past two decades, it has become possible to construct high-density maps for almost all areas of chromosomes using DNA markers based on sequence polymorphisms. Such chromosome maps have become essential tools for linkage analysis of important traits, as well as for genome evolution analysis. Compared to amplified fragment length polymorphism (AFLP) and random amplified polymorphic DNA (RAPD) markers, gene-derived markers are more useful for comparative genomics, and can also serve as phenotype-linked functional markers [1,2].

Grass species are very closely related to one another in comparison to plants in other families [3,4], and as a result, a high degree of similarity is expected in the structure and sequence of grass orthologous genes. Consequently, a great deal of information has been obtained regarding intergenomic synteny and collinearity by using orthologous genes as anchor markers [5,6]. Rice has the smallest genome size among all cereal crops, and much genetic information related to agriculturally important traits has been obtained for this crop. The complete genome of the rice cultivar "Nipponbare" has been sequenced and annotated [7,8], and this data has been used for comparative genomic studies with other grass species.

Common wheat (*Triticum aestivum *L. 2n = 6x = 42, AABBDD) evolved by polyploidization about 10,000 years ago, after which it quickly spread and was domesticated throughout the world [9]. Globally, it is now the most widely cultivated grain, and a large volume of data has been collected regarding genetic factors involved in important traits such as yield, quality and biotic/abiotic stress resistance [10]. Due to both its high agricultural importance and rapid evolution, the level of interest in genomic research on wheat is high. The International Triticeae Mapping Initiative [11] has led a large-scale chromosome mapping project, and data have been organized in the form of the GrainGenes public database [12-14]. Using various grass plant-derived cDNAs as probes, approximately 2,000 RFLP markers have been mapped on linkage maps to date. Furthermore, 6,963 wheat EST sequences have been located on physical maps of wheat by using these ESTs as probes in Southern hybridizations [15,16].

In comparison to RFLP markers, PCR-based markers require less DNA and facilitate high throughput analysis. Thus, the PCR-based marker has become the main tool for genetic analysis. In recent years, numerous PCR-based markers, referred to as "EST-PCR markers", have been developed by designing primers based on EST sequences. Already more than 700,000 wheat EST sequences have been registered with public databases. Most wheat EST-PCR markers were designed from ESTs that contained a simple sequence repeat (SSR) [17-21], and ESTs with SSRs (excluding monomers) are estimated to represent 6.7% of total wheat UniGenes [21]. In order to develop new EST-PCR markers of wheat, it is necessary to make the best use of the abundant wheat EST resources, specifically the remaining 93.3% of UniGenes.

In allohexaploid wheat, PCR products derived from homoeologous genes are often amplified simultaneously. Additionally, if an EST sequence used for marker design is derived from a gene that has paralogues, the number of amplified products is likely to be magnified in a polyploid plant species such as wheat. Such problems hinder the mapping of EST-PCR markers on chromosomes. Recently, sophisticated methodologies for the development of EST-PCR markers for plants have been reported [22-25]. However, in polyploid species, the production of multiple PCR products is unavoidable even when using these methods. For example, Feltus et al. (2006) [23] reported that in the development of EST-PCR markers of orphan crops, up to four products derived from alleles at a particular locus were thought to be produced in an autotetraploid line of *Cynodon dactylon*, and problems were encountered in separating these products. Consequently, when developing EST-PCR markers for wheat, it is essential to eliminate EST sequences derived from paralogous genes and to establish a method to efficiently sort products derived from homoeologous genes.

**Figure 1:**
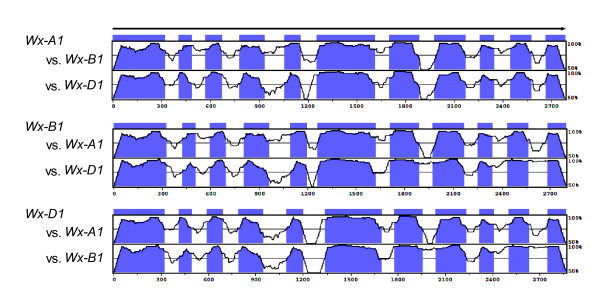
**Comparison of homoeologous *Wx *gene sequences of wheat**. Comparison of the genomic sequences of three homoeologous *Wx *genes [27] using mVISTA [51, 52]. The horizontal axis shows the base number from the start codon for the respective reference gene, while the vertical axis shows the degree of homology. Blue and white regions indicate exon and intron regions, respectively.

Although high degrees of similarity are found among wheat homoeologous genes, intron regions have a greater degree of polymorphism in terms of insertions/deletions and base substitutions than do exon regions [26]. Correspondingly, in the seven sets of wheat genes for which the genomic sequences of the three homoeologous genes are known (*Wx *[27]; *TaHd1 *[28]; *TaDFR *[29]; *wMAD2 *[30]; *Vrn-1 *[31]; *Wknox1 *[32]; *wSSII *[33]), insertions/deletions and base substitutions are more common within intron than within exon sequences (Figure 1). Therefore, if PCR products incorporate intron regions, it may be possible to separate products derived from homoeologous genes using a simple technique such as agarose gel electrophoresis.

When designing primers for EST-PCR markers, primers that span exon-exon junctions in the ESTs prevent amplification of the product from the target gene. To increase the success rate of PCR, a technique for predicting exon-exon junctions in ESTs is required. Based on the assumption that exon regions and exon-intron structures of orthologous genes are highly conserved amongst grass species, we developed an interactive system to design primers in exon regions flanking an intron, using a wheat EST dataset selected by a proprietary program. We confirmed that the primer sets thus designed amplified specific PCR products from target genes, and that the products derived from homoeologous genes could be effectively separated on agarose gels.

## Results

### Establishment of PCR-based landmark unique gene (PLUG) system

**Figure 2:**
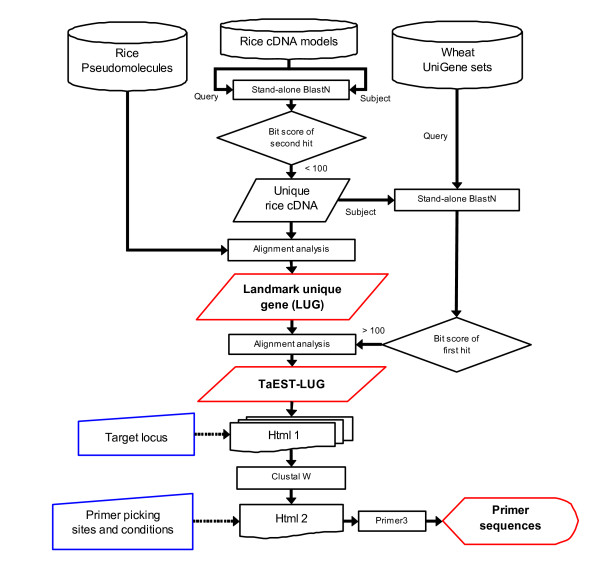
**Flowchart of the PLUG system**. The PCR-based Landmark Unique Gene (PLUG) system extracts primers for wheat by comparing the rice Pseudomolecules database [34] and wheat UniGene data sets [43] in an interactive manner. To eliminate paralogous genes, Landmark Unique Gene loci (LUGs) were selected by pair-wise comparisons of the rice cDNA models [34]. TaEST-LUGs were selected as template loci for potential PLUG markers (see Methods). "Html1" and "Html2" are interactive interfaces where the target locus can be selected and primer picking conditions can be inputted, respectively.

**Figure 3:**
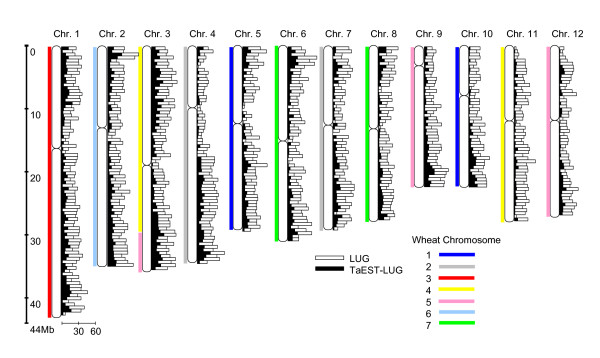
**Distribution of LUGs and TaEST-LUGs on rice chromosomes**. The number of LUGs and TaEST-LUGs for every 500 kb of the rice Pseudomolecules are shown. Rice and wheat synteny, as reported by Gale and Devos (1998) [5] and Sorrells et al. (2003) [35], is shown in different colors for each rice chromosome and wheat chromosome group.

As shown in the flowchart in Figure 2, we developed an interactive system for designing wheat PCR primers. With this system, single-copy genes were extracted by a BLASTN search from among all rice cDNA sequences of the gene models on TIGR Pseudomolecules version 4.0. We defined the loci corresponding to these genes as landmark unique gene loci (LUGs). The system extracted a total of 17,130 LUGs, which account for 30.6% (17,130/55,890) of the all predicted gene loci [34], and there were 5,665 LUGs with high homology to wheat ESTs in the UniGene databases. From these 5,665 LUGs, 4,312 TaEST-LUGs were selected as template loci for potential PLUG markers, as they reached our designated minimum length threshold and spanned an area that included an intron(s) in the corresponding rice gene (TaEST-LUGs are shown in Additional File 1). The number of TaEST-LUGs accounted for 25.2% of the LUGs or 7.7% of the total rice gene loci. Figure 3 shows the distribution of LUGs and TaEST-LUGs for each rice chromosome (complete data shown in Additional File 2). Higher densities of LUGs and TaEST-LUGs were generally observed in the distal as opposed to the proximal regions of the chromosome arms. The numbers of TaEST-LUGs also varied greatly among chromosomes: high on chromosomes 3 and 1, but low on chromosomes 11 and 12. The number of TaEST-LUGs on chromosome 3 was more than five times that for chromosome 11 (see Additional File 2).

To estimate the number of potential PLUG markers on each wheat chromosome group, TaEST-LUGs were assigned to wheat chromosomes based on previously reported data for rice and wheat synteny [5,35]. As an example, rice chromosomes 11 and 12, which have fewer TaEST-LUGs, showed synteny with regions of the wheat chromosome groups 4 and 5, respectively (Figure 3). However, wheat chromosome group 4 also corresponds to a large part of rice chromosome 3, while group 5 corresponds to the remaining part of chromosome 3 and all of chromosome 9. Therefore, the distribution bias of TaEST-LUGs on wheat chromosome groups is lower than that for rice chromosomes, and as a result, the number of potential PLUG markers per wheat chromosome group was estimated to be 450–650 (data not shown).

### PCR amplification with PLUG primers

To demonstrate the performance of the PLUG system, one marker was randomly selected from the short and long arms of each of the 12 rice chromosomes, giving a total of 24 markers. To design PCR primers for these markers, the following settings were used for the PLUG system: Melting temperature was 55–65°C (optimum: 60°C), primer length was 18–25 bases (optimum: 21), and the desired size of amplified fragments estimated based on the rice genome was approximately 1 kb (Table 1).

**Table 1:** Primer sets designed by the PLUG system

			Primer sequence (5' → 3')		Estimated product size	
				
Marker no.	TIGR Rice locus ID	TaEST clone	Forward	Reverse	Os genomic (bp)	Ta EST (bp)
1	LOC_Os01g07960	AY093953	agtacgggaggacgcatgt	tctgcaggttcggtagacaat	1128	297
2	LOC_Os01g62430	BT009397	cttcggcagcgatttccta	gtgaacgtgaggcctactctg	868	353
3	LOC_Os02g01440	CD453605	ccaccacagaagcagatgaat	gctagatggcacaccaagtg	844	208
4	LOC_Os02g49780	CK207954	aacaagatggcgaggaagaac	agaactcagatgcaggctcaa	869	216
5	LOC_Os03g03510	CK162308	gtcaagatcgccaaggacac	gcctccctcaacaaactcaag	1049	213
6	LOC_Os03g48000	CK158455	aatgcatgttgaacctcgtgt	tcaaggagatcgatgagcatt	963	156
7	LOC_Os04g08350	CA486283	acctcacctcatcactggaaa	attgcttcagcctcctttctc	1030	296
8	LOC_Os04g41910	CD913720	gagaggaatgcgtgaagtttg	agaccatctttccggtctttg	1031	106
9	LOC_Os05g01240	CK162649	tttccgcttcctatgatgcta	ttcccatctcttgccattaaa	765	340
10	LOC_Os05g28200	CK168220	gggatagaactctgggacttca	agtgccagggcataatacagc	937	147
11	LOC_Os06g13680	CK214580	ctttagcctccttcgcaacat	tcctcatggttctcaagcact	1069	89
12	LOC_Os06g46450	CK162440	tttcacaggaacctctgcatc	tcaacatttgcaggattgtca	765	150
13	LOC_Os07g16960	CD918004	acgtgtgcgacttgaagagat	acagcttgctgcttccagaat	836	171
14	LOC_Os07g30840	DR737909	cgtgctaactttggctgagtc	gcactcgttgatgaggaaatc	842	108
15	LOC_Os08g05890	CK206352	Gccagtttcctcgagatcc	cacagtactgctttgggttgg	990	144
16	LOC_Os08g44000	CK161204	gcaatatgcggtgcctatact	cccagccagtctctcacataat	971	207
17	LOC_Os09g04800	CK162348	cggctacaataacggtgactc	ctctgctgatctgaaggatgg	996	322
18	LOC_Os09g36450	CK162719	Ttcttggtcactctgagcgta	ttgctagctcagcacagtttg	1007	389
19	LOC_Os10g17280	DN949140	agccattcacagctcttcttg	aatatgcttcctggagtcacg	897	226
20	LOC_Os10g32880	CK210932	tcatcgagcgctacattgag	ttgtcttgctgtgtgaagctg	1055	217
21	LOC_Os11g06340	CK212529	acccgttgatcccaagaagta	cggtatcatcagcctcaactc	923	106
22	LOC_Os11g38020	CA680245	agcaacactggaggagatatcag	ccattccaaccttatgtatgtca	880	358
23	LOC_Os12g13390	CK207363	ctcctcggaaggtctcaagat	tacaacgcttggttgggtatc	1057	237
24	LOC_Os12g35270	BJ227772	gctacaacccggcactcat	tggtgcttcttcgacttcttg	998	88

One TaEST-LUG was randomly selected from each arm of the 12 rice chromosomes, and from these loci, 24 PLUG primer sets were produced. The table shows the TIGR rice locus ID, the accession numbers of wheat ESTs exhibiting homology (scores > 100), the sequences of forward and reverse primers, the size of the region flanked by the primers along the rice genomic sequence, and the size of the region flanked by the primer along the wheat EST sequence.

**Figure 4:**
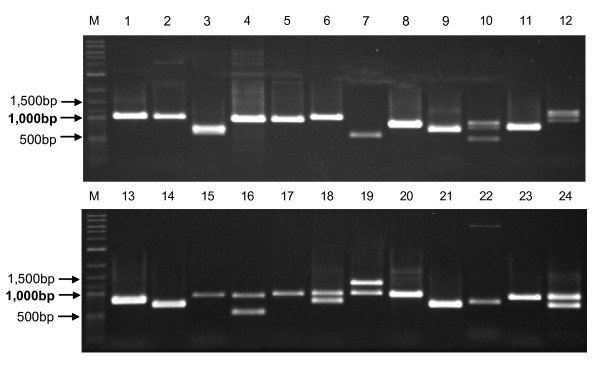
**1% agarose gel electrophoresis of PCR products**. PCR products derived from 24 PLUG primer sets were separated using a 1% agarose gel in TAE buffer. Lane numbers correspond to marker numbers indicated in Table 1. M: 2-Log DNA Ladder (New England BioLabs Inc., Ipswich, MA, USA).

PCR was performed with the 24 primer sets using genomic DNA from the wheat variety Chinese Spring as a template. Electrophoresis on a 1% agarose gel clearly separated the PCR products into one to three bands: three bands were obtained with two primer sets (No. 10 and 12) (Type I), two bands with five primer sets (No. 3, 16, 18, 19 and 24) (Type II), and a single band with the remaining 17 primer sets (Type III) (Figure 4). The size of these products ranged from 500 to 1,500 bp, and each product was larger than the size predicted from the wheat EST (Table 1), suggesting that all PCR products contained introns.

With Type I primer sets, it is likely that the three products corresponded to products derived from three homoeologous genes. With Type II and III primers, products from more than one gene were likely present in some of the bands detected in the 1% agarose gel.

### Assignment of PLUG markers to wheat chromosomes

To assign the 24 PLUG markers to wheat chromosomes, PCR products obtained from nullisomic-tetrasomic lines were either separated on 1% agarose gels, or were digested with either *Hae*III or *Taq*I restriction enzyme then separated on 4% agarose gels.

**Figure 5:**
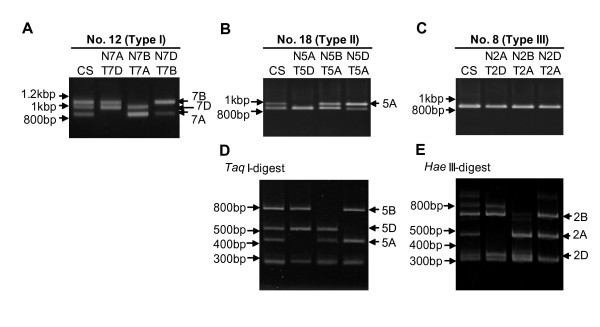
**Nullisomic-tetrasomic analysis of PLUG markers**. PLUG markers were assigned to wheat chromosomes by the presence or absence of PCR products from nullisomic-tetrasomic lines. A-C: 1% agarose gel electrophoresis of the PCR products of Marker No. 12 (A), Marker No. 18 (B), and Marker No. 8 (C). D and E: 4% agarose gel electrophoresis of the *Taq*I-digested products of Marker No. 18 (D) and *Hae*III-digested products of Marker No. 8 (E).

With Type I Marker No.10, we determined that the three bands obtained were amplified from chromosomes 1A, 1B and 1D, while with Type I Marker No. 12, products were from chromosomes 7A, 7B and 7D (Figure 5A, Table 2).

**Table 2:** Chromosome locations and putative annotations of the PLUG markers

		Wheat chromosome			
			
Marker no.	Type	undigested, 1% agarose	*Hae *III-digest, 4% agarose	*Taq *I-digest, 4% agarose	Annotation of orthologous rice gene (Pseudomolecules ver. 4)
1	III			3A, 3B, 3D	Phospholipase/Carboxylesterase family protein
2	III		3A, 3B	3A, 3B	Elicitor-responsive protein 1, putative
3	II	6B	6B	6A, 6B, 6D	GTP-binding protein, putative
4	III			6B, 6D	expressed protein
5	III		5A, 4D	5A, 4D	CIPK-like protein 1, putative
6	III				magnesium transporter CorA-like family protein, putative
7	III				Cysteine synthase, chloroplast precursor, putative
8	III		2A, 2B, 2D	2B	RNA recognition motif family protein
9	III			1A, 1B, 1D	AML6, putative
10	I	1A, 1B, 1D	1A, 1B, 1D	1A	chlorophyll synthase, ChlG family protein
11	III		7A, 7D		senescence-associated protein, putative
12	I	7A, 7B, 7D	7A, 7B, 7D	7A, 7B, 7D	Polyprenyl synthetase family protein
13	III			3A, 5A, 5D	Phosphatidylinositol N-acetylglucosaminyltransferase subunit A, putative
14	III				COP9 signalosome complex subunit 7, putative
15	III		7A	7B, 7D	MSP domain containing protein
16	II	7B		7B	expressed protein
17	III			7A	BadF/BadG/BcrA/BcrD ATPase family protein
18	II	5A	5A, 5B	5A, 5B, 5D	Triosephosphate isomerase, chloroplast precursor, putative
19	II	1A	1B, 1D	1A, 1B, 1D	ATP synthase gamma chain, mitochondrial precursor, putative
20	III		1A	1A	PRP19/PSO4 homolog, putative
21	III				Ubiquinol-cytochrome c reductase complex 7.8 kDa protein, putative
22	III				Small GTP-binding protein domain containing protein
23	III		5A	5B	Aspartyl aminopeptidase, putative
24	II	5B	5A, 5B, 5D	5A, 5B	expressed protein

Types I, II and III indicate that 1% agarose gel electrophoresis of PCR products resulted in the separation of three, two, or single bands, respectively. PLUG markers were assigned to chromosomes by electrophoresis on 1% agarose gels, or by electrophoresis of *Hae*III- or *Taq*I-digested fragments on 4% agarose gels. The table also shows the annotations of TaEST-LUGs that were used for marker development.

For Marker No. 18 (Type II), the longer band on the 1% agarose gel was the product derived from chromosome 5A (Figure 5B). After separating *Taq*I-digested products on a 4% agarose gel, we confirmed that the shorter band contained two products derived from chromosomes 5B and 5D (Figure 5D). For the other Type II markers, we identified three chromosomes for Marker No. 3, 19 and 24, and a single chromosome for Marker No. 16 (Table 2).

According to PCR-RFLP analyses, the single band detected on a 1% agarose gel with Marker No. 8 consisted of the products derived from chromosomes 2A, 2B and 2D (Figure 5C and 5E). Similarly, for the other Type III markers, we determined that four markers (No. 1, 9, 13 and 15) could be assigned to three chromosomes, five markers (No. 2, 4, 5, 11 and 23) to two chromosomes, and two markers (No. 17 and 20) to a single chromosome (Table 2).

In total, 19 of the 24 markers were assigned to at least one chromosome.

### Sequence comparison of the PLUG markers

To confirm that the 24 markers were derived from the target genes, the PCR products were cloned and sequenced. Sequences of the clones were compared to the wheat EST sequences used for primer design, confirming that all markers were derived from the target genes and contained at least one intron. The sequences of the exon regions were compared to the EST sequences, and very high degrees of similarity (> 95%) were observed.

Five of the markers contained two related but not identical sequences, while 18 of the markers contained three related sequences. Marker No. 13 was unusual in that five related sequences were found among the clones derived from this product (see Additional File 3). For all markers, sequencing of products confirmed that *Hae*III or *Taq*I restriction sites occurred at the appropriate positions to result in the restriction fragment size polymorphisms that were used to assign markers to chromosomes (Table 2).

**Figure 6:**
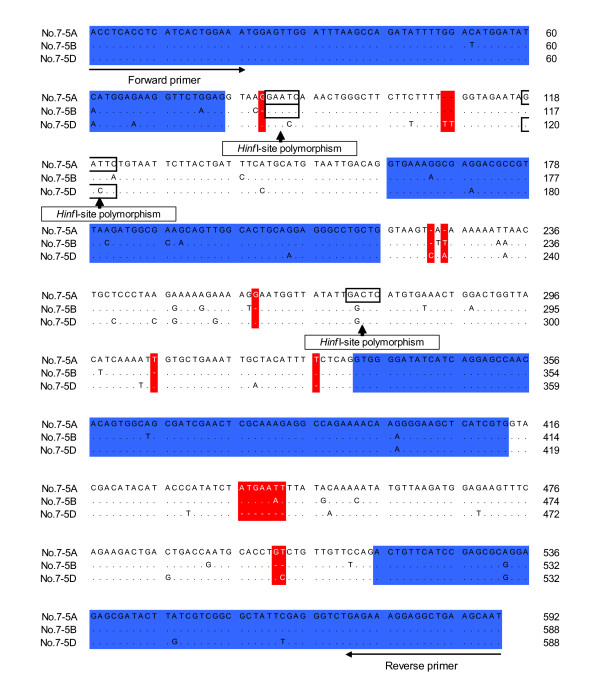
**Sequence alignment of Marker No. 7**. Dots indicate identical bases among sequences, while dashes indicate deletions (red). Blue areas indicate exons as estimated by the alignment of the EST sequence and genomic sequences. PCR-RFLP analysis using three *Hinf*I site polymorphisms allowed Marker No. 7 to be assigned to chromosomes 5A, 5B and 5D (data not shown).

For Marker No. 7, which could not be assigned to a chromosome, the three sequences obtained were compared in a pair-wise manner. The degrees of similarity between pairs ranged from 95 to 97% in the exon regions and from 88 to 92% in the intron region. These sequence alignments also indicated that insertion/deletion polymorphisms existed in the intron regions, but not in the exon regions (Figure 6). Using *Hinf*I recognition site polymorphisms, the marker could be assigned to chromosomes 5A, 5B and 5D. For each of the 24 primer sets, similarity between sequences derived from homoeologous genes was lower in the intron regions (88% on average) than in the exon regions (average 98%). It is notable that all insertion/deletion polymorphisms among homoeologous gene sequences were found in the intron regions.

## Discussion

Gene expression data indicates that in rice, about 48% of all predicted genes are expressed, based on the ratio of expressed transcriptional units (TU) to total TU (Knowledge-based Oryza Molecular biological Encyclopedia [36]). According to this proportion, the number of LUGs corresponding to expressed genes is approximately 8,000, whereas in this study, a total of 5,665 LUGs showed high homology to available wheat ESTs. Since the wheat UniGene datasets consist of numerous EST sequences derived from multiple libraries, and are therefore considered to cover most expressed genes, the difference in these numbers is thought to be primarily due to sequence diversity between wheat and rice, rather than the number of available wheat UniGenes. Therefore, using the same BLASTN search threshold levels as were used in this study, a significant increase in the number of LUGs with homology to wheat ESTs is not likely to be observed in the future. In contrast, improvements in cDNA sequence databases would result in an increase in the proportion of these LUGs that could be used for marker development. The availability of sequences from a wheat full-length cDNA library, for example, should result in a large increase in TaEST-LUGs.

The PLUG markers were produced from 24 randomly selected TaEST-LUGs, yet all of them amplified specific products. Therefore, when designing primers from TaEST-LUGs, quite a high success rate can be expected. Our success rate was higher than rates achieved with pearl millet and sorghum EST-PCR markers based on the rice genome database [23,37]. For example, Feltus et al. (2006) [23] compared the rice genome and sorghum UniGenes and prepared 384 conserved intron-spanning primer (CISP) sets. These CISP sets achieved a success rate of 81%. A high success rate was observed in our study because, unlike the automatic pipeline systems for the CISPs, the PLUG system allows PCR product length and primer picking condition data to be entered independently (Figure 2). This manual procedure enables us to avoid areas close to exon-exon junctions where sequences can be ambiguously aligned, and also allows us to identify highly conserved regions. In addition, an optimal primer set can be selected from the three candidate primer sets recommended by the system. Furthermore, by adjusting the position of the primer, the size of the expected product can be set from 500 to 5,000 bp. This flexibility is useful in redesigning primers to amplify different regions of the TaEST-LUGs.

In wheat, EST-PCR markers often yield multiple products originating from paralogous genes, as well as from homoeologous genes. Thus, we developed a system based on rice single-copy gene loci under the hypothesis that the copy number of genes was highly conserved between rice and wheat. This hypothesis appears to be correct, as in the majority of cases the number of products amplified with the PLUG primers was only two or three. In addition, sequence analysis confirmed that PLUG amplification products were derived from target genes and contained introns in the expected positions. The genes represented by TaEST-LUGs were highly conserved between wheat and rice, not only in terms of copy number but also in sequence and exon-intron structure, and the PLUG markers appeared to represent the wheat orthologs of the genes on the TaEST-LUGs.

Nullisomic-tetrasomic analysis indicated that some amplification products could not be clearly separated and assigned to chromosomes using the basic methods of this study. However, comparisons between homoeologous sequences allowed us to detect insertions/deletions and base substitutions related to recognition site polymorphisms other than *Hae*III and *Taq*I. Therefore, by using additional restriction endonucleases in the PCR-RFLP analysis, it should be possible to assign all sequences to chromosomes. Indeed, using *Hinf*I, Marker No. 7 was assigned to the group 5 chromosomes (Figure 6). Furthermore, sequence analysis of all twenty-four markers revealed that while exon regions were generally equal in length, intron regions had insertion/deletion polymorphisms every 100 bp on average (data not shown). This suggests that products derived from homoeologous genes can be sorted based on differences in length, without resorting to sequencing or RFLP analysis. Generally, to detect slight size differences in PCR products, high-resolution electrophoresis, which is both time-consuming and expensive, is required. However, in recent years, a high-resolution low-cost capillary electrophoresis device has become available [38]. Such a device may enable high throughput analysis, which would be required for the development of genome-wide PLUG markers. Once markers are assigned to chromosomes, a series of deletion lines [39] is available for determining marker locations within the respective chromosomes.

Nineteen of the 24 markers could be assigned to at least one chromosome by the simple detection methods of either electrophoresis on a 1% agarose gel or restriction digestion with *Hae*III or *Taq*I followed by electrophoresis on a 4% agarose gel. Sixteen markers were assigned to the same chromosomes as was predicted by the previously reported synteny data for rice and wheat [5,35]. For example, synteny has been reported between rice chromosome 1 and wheat chromosome group 3, and in the present study, two markers (No. 1 and 2) that were produced based on TaEST-LUGs from rice chromosome 1 were located on wheat chromosomes 3A, 3B, and 3D (Table 2). Marker No. 5, which was located on chromosomes 5A and 4D, was developed based on the TaEST-LUG in the short arm of rice chromosome 3, which corresponds to wheat chromosome group 4. Therefore, it can be assumed that Marker No. 5 is located on a known reciprocal translocation region between 4A and 5A [40]. These results indicate that the synteny between rice and wheat facilitates the effective development of EST-PCR markers on targeted wheat chromosomes. On the other hand, Markers No. 7 and 17 were predicted to be located on chromosome groups 2 and 5, respectively; however, they were actually located on chromosome group 5 and chromosome 7A, indicating some synteny perturbation between rice and wheat in these areas. Furthermore, Marker No. 13 was predicted to be located on chromosome group 2, but it was actually located on 3A, 5A, and 5B chromosomes. Further investigation revealed that the marker was also located on 3B and 5D chromosomes (data not shown). This suggests that Marker No. 13 is derived from paralogous genes resulting from a gene duplication event in the wheat genome; furthermore, these genes are located in regions where the synteny between rice and wheat is perturbed.

The EST sequences of the PLUG markers were used as queries in BLASTN searches of the GrainGenes [14] databases. Results indicated that 10 of the 24 EST sequences showed high similarity with bin-mapped ESTs, three ESTs showed high similarity with EST-SSR sequences, and three ESTs showed high similarity with sequences in the sequenced probe database (see Additional File 3), with some marker sequences being present in more than one of these databases. Totally 12 out of 24 markers were based on ESTs that have not previously been used for marker development. Of the remaining 12 markers, 11 markers are derived from genes that were assigned to wheat chromosomes in previous studies [15,16,41]. No contradictions in the assigned chromosomes were observed between this study and previous studies; however, the numbers of chromosomes the corresponding genes were assigned to varied (see Additional file 3). For example, in a previous bin mapping study, the gene corresponding to Marker No. 1 was located on chromosome 3B; however, in this study, it was located on chromosomes 3A, 3B, and 3D. Hence, mapping information was further enriched with mutually complementary data. In summary, by designing primers from the approximately 4,000 TaEST-LUGs extracted using the system, it would be expected that 3,000 markers could be assigned to at least one chromosome by the same methods used here, and that 1,500 of these markers would be novel. Furthermore, even if the markers developed are not new, they may be useful for enriching mapping information.

In this study, we showed that the PLUG system could be used to develop new markers on specifically targeted wheat chromosomes by taking advantage of synteny with rice chromosomes. This implies that the PLUG markers can accurately show the chromosomal locations of wheat genes that are orthologous to rice genes, and that the markers can act as scaffolds for comparative genomics. Furthermore, locus specific sequences can be readily obtained from the PLUG markers, and by utilizing PLUG markers as probes, it will be possible to quickly identify target clones from the huge BAC library for wheat. The locus-specific sequences obtained from PLUG markers are also potentially very useful for surveying sequence polymorphisms among wheat cultivars, allowing the development of new markers near quantitative trait loci (QTLs). We are presently using the TaEST-LUGs to develop PLUG markers for the entire wheat genome and to carry out bin mapping. A web site is under construction to allow public access to the PLUG system.

## Conclusion

With the PLUG system, 4,312 TaEST-LUGs were shown to be useful as reliable standards for wheat EST-PCR marker development. In a study using 24 randomly selected TaEST-LUGs, half showed high homology to wheat EST sequences that have not previously been used for marker development. Therefore, by carrying out large-scale wheat marker development using the PLUG system, we can potentially double the number of gene-derived markers.

In comparisons of intron sequences from wheat homoeologous genes, insertion/deletion polymorphisms were found in almost all cases. This suggests that the sorting of multiple PCR products derived from homoeologous genes, which is a major block for EST-PCR marker development, can be resolved by including an intron sequence in PCR products. Therefore, the PLUG system, which semi-automatically extracts primer sets flanking an intron sequence, is a very effective tool in wheat marker development. With this system, it is now possible to sort homoeologous genes using a low-cost and convenient separation method, thus allowing large-scale PCR-based marker development for wheat.

Since the PLUG system is based on orthologous gene conservation, markers produced using the system can also be used as accurate anchor markers for genomic research comparing rice and wheat. Furthermore, PLUG markers can show the positional relationships of wheat homoeologous genes, and as a result, these markers are expected to contribute greatly to research on synteny dissociation among wheat homoeologous chromosomes.

## Methods

### Wheat genetic information

All primers were designed from the non-redundant EST sequences in the wheat UniGene database managed by NCBI [42,43]. There were 38,566 non-redundant sequences after processing the 743,872 ESTs in wheat UniGene #46 (July 2006).

### Rice genetic information

Wheat gene structures (exon-intron junction sites) were predicted by alignment assembly between 12 rice contig sequences (Pseudomolecules) assembled with a minimum tiling path of 3,408 BAC/PAC clones and rice cDNA models containing the untranslated region but no intron sequences. Annotations for cDNA were obtained through the rice genome annotation database Osa1 [44]. All data were downloaded from TIGR ftp sites [45].

### Homology and alignment analysis

The system for designing wheat EST-derived primers is shown in the flow chart in Figure 2. In the first step, all cDNA sequences on rice Pseudomolecules were compared using a local BLASTN (stand-alone BLAST) program to search for the single-copy genes. When the second hit value was 100 or more, the queried cDNA was eliminated from subsequent data analyses as a multi-copy gene. The loci corresponding to single-copy cDNAs were defined as landmark unique gene loci (LUGs). In the second step, the cDNA sequences of the LUGs were compared to the annotated gene models (transcriptional units) in the Pseudomolecules database using BLASTN, and each aligned HSP (high-scoring segment pair) position was considered a putative exon region. No BLAST match positions within rice gene models were assumed to be intron regions or low-complexity regions unsuitable for primer design. The wheat UniGene set was matched against the rice single-copy cDNA to select candidate orthologous ESTs meeting the similarity threshold (Score > 100). Finally, to predict exon-exon junctions, wheat EST sequences were aligned with corresponding rice cDNA and genomic sequences using the CLUSTAL W program [46].

From the candidate orthologous wheat ESTs, we selected ESTs for potential PLUG markers according to the following criteria: the EST had to contain at least a single predicted exon-exon junction, show successive > 40 bp conserved sequences with rice cDNA in both adjacent regions of the exon-exon junction, and incorporate > 30 bp of intron sequence within the exon-exon junction predicted from the rice genomic sequence. TaEST-LUGs were defined as the LUGs that showed high homology with the selected ESTs based on these criteria.

### Primer picking protocol

Interactive html files for picking primers were produced from interface codes and multiple alignment data on the rice locus with a single-copy cDNA model. The input interface requires the product size range, Tm (minimum, maximum, and optimum), and primer length (minimum, maximum, and optimum), as well as the start and end points of multiple alignment data for the rice gene model, rice cDNA and wheat EST sequences. However, accurate target positions on exons need not be input for our software to automatically design primer sequences from the wheat EST regions aligned with the intron-spanning exons in the rice gene structure within the approximate input range. Primer sequences are designed using Primer3 software [47] with entered conditions. Along with primer sequences, the predicted length of the PCR product in rice, the reverse-complementary primer sequences, and the Tm value are also displayed in output data.

### Plant materials and DNA extraction

Seeds of wheat (*Triticum aestivum *cv. Chinese Spring) and a set of Chinese Spring nullisomic-tetrasomic lines [48] were obtained from the John Innes Center Public Collection. In this study, 21 nullisomic-tetrasomic lines lacking each pair of the 21 homologous chromosome pairs were used to determine the location of markers. Genomic DNA was extracted from 100 mg of young leaf tissue using the Nucleon PhytoPure Plant DNA Extraction Kit (Amersham Biosciences, Little Chalfont, UK) according to the manufacturer's instructions.

### Molecular analysis

PCR amplification of genomic DNA was carried out using the primer sets designed by the PLUG system (Table 1). Each 25-μL PCR reaction mixture included 50–100 ng of DNA, 1.5 mM MgCl_2_, 5 pmol of each primer, 0.2 mM dNTP (each), 1 × *Ex Taq *buffer, and 0.5 U of *Ex Taq *polymerase (Takara, Osaka, Japan). The PCR cycle consisted of an initial 5 min denaturation at 95°C, followed by 32 cycles of 95°C for 30 s, 58°C for 30 s, and 72°C for 2 min, and final extension at 72°C for 7 min. PCR was conducted using a GeneAmp PCR system 9700 (Applied Biosystems, Foster city, CA, USA). An 8-μL aliquot of the PCR mixture was analyzed by electrophoresis on a 1% agarose gel in 40 mM Tris – acetate – 1 mM EDTA (TAE) buffer.

For PCR-RFLP analysis, an 8-μL aliquot of the mixture was digested overnight with 2.0 U of *Hae*III or *Taq*I in incubators set at 37 or 65°C, respectively. *Hae*III and *Taq*I are relatively inexpensive endonucleases with 4-bp recognition sites. Therefore, they are frequently used in surveys of sequence polymorphisms in PCR products [49,50]. Digested fragments were fractionated by electrophoresis on a 4% agarose gel in TAE buffer. Band sizes were estimated against a '2-Log DNA Ladder' (New England BioLabs Inc., Ipswich, MA, USA).

For each primer set, PCR products from the genomic DNA of Chinese Spring were separated by electrophoresis on a 1% agarose gel, then excised from the gel and purified using a QIAquick Gel Extraction Kit (QIAGEN, Hilden, Germany). Purified products were cloned using the TOPO TA cloning kit (Invitrogen, Carlsbad, CA, USA). Inserts of 6–12 clones for each primer set were sequenced using a CEQ8000 DNA analysis system (Beckman Coulter, Inc., Fullerton, CA, USA).

## Authors' contributions

GI did wet-lab testing of a subset of primer pairs, and drafted the manuscript. JY did all programming and design of computational experiments and databases, and helped to draft the manuscript. MS participated in the design of the study and performed sequence analyses. TN conceived of the study, and participated in its design and coordination and helped to draft the manuscript. All authors read and approved the final manuscript.

## Supplementary Material

Additional file 1 **List of TaEST-LUGs**. TIGR rice locus IDs for the TaEST-LUGs extracted by the PLUG system are listed. Click here for file

Additional file 2 **Numbers of LUGs and TaEST-LUGs**. The worksheet shows the number of loci, LUGs, and TaEST-LUGs per 500 kb on each rice chromosome of the TIGR Pseudomolecules. Click here for file

Additional file 3 **Detailed data for 24 PLUG markers**. The following data for the 24 PLUG markers are shown: TIGR rice locus ID, annotation of the rice locus, ID of wheat UniGene, accession number of the longest EST, sequences of forward and reverse primers, estimated sizes of rice and wheat PCR products, wheat PCR product sizes (assigned chromosome) and their *Hae*III/*Taq*I-digested fragment sizes (assigned chromosome), results of BLASTN searches of the GrainGenes [14] databases, and results of sequence analysis. Click here for file
